# 309. Optimized hematopoietic stem cell source for the *ex vivo* production of mature human neutrophils as a potential cell-based therapy for neutropenia

**DOI:** 10.1093/ofid/ofad500.381

**Published:** 2023-11-27

**Authors:** Kyle D Timmer, Daniel Floyd, Nathan E Jeffries, Emma Yvanovich, David B Sykes, Michael Mansour

**Affiliations:** UMass Chan School of Medicine, Worcester, Massachusetts; Massachusetts General Hospital, Somerville, Massachusetts; Massachusetts General Hospital, Somerville, Massachusetts; Massachusetts General Hospital, Somerville, Massachusetts; MGH, Boston, Massachusetts; Massachusetts General Hospital, Somerville, Massachusetts

## Abstract

**Background:**

Neutrophils or granulocytes are the most common innate immune cell and are critical for eliminating invasive pathogens. Patients with low neutrophil counts face an increased risk of fatal opportunistic infections. Allogeneic granulocyte transfusions offer a solution to supplement endogenous neutrophil counts but are challenging due to insufficient preservation methods and donation feasibility. Here, we compare the capacity of three different hematopoietic stem cell (HSC) sources; 1) cord blood (CB), 2) live adult bone marrow (LABM), and 3) cadaveric adult bone marrow (CABM), to expand and generate an *ex vivo* neutrophil source.

**Methods:**

HSCs were expanded *ex vivo* with or without UM729 then differentiated and evaluated for neutrophil yield and purity. *Ex vivo* differentiated neutrophils were characterized by flow cytometry for activated phenotype surface markers. Finally, the functional capacity of *ex vivo* neutrophils, including phagocytosis, ectodomain shedding, and reactive oxygen species (ROS) production, was measured in response to the fungal pathogen *Candida albicans*.

**Results:**

CB-derived HSCs were found to be superior in expansion capacity compared to LABM- or CABM-derived HSCs by 3-fold at day six. The use of UM729 reduced the purity of neutrophil differentiation by 44% for both live and deceased BM marrow-derived HSCs. UM729 had no observable effect on the purity of neutrophil differentiation for CB-derived HSCs. Additionally, due to enhanced Fc gamma receptor I expression, *ex vivo* neutrophils from all sources were ten times more sensitive to recognizing IgG-opsonized *C. albicans* than primary neutrophils. *Ex vivo* neutrophils were capable of phagocytosis, ROS production, and ectodomain shedding to a similar capacity as primary donor neutrophils.

**Conclusion:**

We find that cadaveric bone marrow-derived HSCs are a viable source for HSC-generated neutrophils. That said, CB-derived HSC were superior in expansion capacity. We also highlight the unique opsonin-activating features of *ex vivo* generated neutrophils which warrant further study in granulocyte transfusion animal models of systemic infection. These data underscore the significance of neutrophil cellular therapeutics as a novel modality for preventing infection in high-risk neutropenic patients.

**Disclosures:**

**Nathan E. Jeffries, BA**, Safi Biotherapeutics: Salary **Michael Mansour, MD, PhD**, Thermofisher: Grant/Research Support

